# Personality and metabolic scope in wild mice

**DOI:** 10.1242/jeb.250374

**Published:** 2025-08-11

**Authors:** Wyeth Blumberg, Alyssa Fiedler, Vincent Careau

**Affiliations:** Department of Biology, University of Ottawa, Ottawa, ON, Canada, K1N 9A4

**Keywords:** Energetics, Aerobic capacity, Novel environment, Basal metabolic rate, Sampling bias, Forced exercise

## Abstract

It is intuitive to expect a relationship between animal personality (i.e. consistent individual behavioural differences) and metabolic rate, but the literature contains mixed results. Most studies have measured resting metabolic rate (RMR); yet, other metrics such as *V̇*_O_2_,max_ and metabolic scope may also relate to personality. Here, we explored the relationships between personality (docility and exploration) and three metabolic traits (RMR, *V̇*_O_2_,max_ and metabolic scope) in wild mice (*Peromyscus leucopus*). We found no among-individual correlation (*r*_ind_) between personality and motivation to run during *V̇*_O_2_,max_ trials, suggesting that using our standard forced-exercise test did not introduce a personality-related sampling bias. At the within-individual level, we found a positive and significant relationship between docility and metabolic scope, and the correlation was entirely driven by *V̇*_O_2_,max_. Finally, we found a positive and significant *r*_ind_ between RMR and time spent grooming during the open-field test, which may be caused by the stress response.

## Teaser

**Highlighted Article:** A forced-exercise test in *Peromyscus leucopus* does not introduce a personality-related sampling bias; a positive relationship exists between resting metabolic rate and time spent grooming during an open-field test.

## INTRODUCTION

Consistent individual variation in behaviour across time and situations, referred to as personality (Réale et al., 2007), has received considerable interest over the last few decades (Sih et al., 2004; Dingemanse et al., 2010; Laskowski et al., 2022). Part of this growing interest lies in better understanding trait integration, in which correlational selection favours specific trait combinations that are required to optimally accomplish fitness-enhancing functions (Pigliucci and Preston, 2004). Trait integration is interesting to study not only among personality traits but also with other aspects of the phenotype, including life-history, hormonal, immune, performance and metabolic traits (Réale et al., 2010; Careau and Garland, 2012). The potential for integration between animal personality and metabolic traits is high because the behaviours that make up personality bear direct and indirect consequences on energy turnover (Careau et al., 2008; Biro and Stamps, 2010; Careau and Garland, 2012). In other words, individuals with different personality types may have different energetic input and expenditure, which, in turn, may be a cause or consequence of individual differences in resting metabolic rate (RMR; the metabolic rate of a postabsorptive organism at rest within its thermoneutral zone). Because RMR excludes any form of locomotor activity (by definition), behaviour cannot directly contribute to variation in RMR, making any observed relationship between personality traits and RMR interesting but relatively puzzling to explain.

Two main models – the performance and allocation models – were proposed to explain how variability in RMR may cause or arise from the different way that animals manage their energy budget (Bennett and Ruben, 1979; Nilsson, 2002), and Careau et al. (2008) specifically applied these models to the study of personality. In the performance model, a high RMR reflects the maintenance cost of the large metabolic machinery needed to sustain high levels of activity; RMR should thus be positively correlated with energetically costly behaviours. By contrast, the allocation model predicts a negative relationship due to finite resources that must be allocated between processes leading to higher RMR and activity. Although data from various artificially selected lines and strains initially provided strong support for the performance model (Biro and Stamps, 2010), studies at the within-population level provided modest overall support for the performance model (Biro and Stamps, 2010; Mathot et al., 2019). However, most of the relationships were tested at the phenotypic level, which may be misleading when trying to relate such highly labile phenotypes as behaviour and metabolism (Dingemanse and Dochtermann, 2013). It is therefore particularly important to collect paired repeated measurements and use (co)variance partitioning to test the relationship at the among- and within-individual levels (e.g. Abdeen et al., 2022).

While most studies have considered RMR (but see Careau et al., 2015), other metabolic metrics could also be relevant to understanding the relationship between personality and metabolic rate. For example, Careau and Garland (2012) raised *V̇*_O_2_,max_ as worth considering in the suite of metabolic traits that might covary with behaviour. Moreover, metabolic scope (=*V̇*_O_2_,max_−RMR) has been suggested to be ‘equally (or more)’ relevant to behaviour as RMR (Biro et al., 2018). This is argued because metabolic scope represents the lower and upper bounds on behavioural activities, and Biro et al. (2018) used this to make predictions of frequency distributions for behavioural activity over longer intervals. Thus, a higher metabolic scope could mean a higher spare energy reserve for intense but non-maximal activity. In support of this idea, fish with higher metabolic scope also had higher levels of aggression and dominance behaviour (Killen et al., 2014), mouse lines bred for greater voluntary wheel running had higher metabolic scope than control lines (Biro et al., 2018), and wild voles with greater metabolic scope displayed greater activity and home range (Boratyński et al., 2020). Other than these studies, we are not aware of any empirical support for a relationship between metabolic scope and personality traits.

Because metabolic scope requires measurement of *V̇*_O_2_,max_ using forced-exercise tests, it introduces the additional challenge of motivating the subjects to exert at their maximum, which may generate personality-related sampling bias in *V̇*_O_2_,max_ measurements (Careau et al., 2008; Biro and Dingemanse, 2009). In a previous study on wild white-footed mice (*Peromyscus leucopus*), we showed that individuals consistently differed in their ‘willingness to run’ during a forced-exercise test designed to measure *V̇*_O_2_,max_ (Fiedler and Careau, 2021). Fiedler and Careau (2021) used data from a larger project that also involved measurements of repeatable behavioural traits: docility in a bag test and activity in an open-field test (i.e. exploration). Therefore, there is an opportunity to fill an existing gap in the literature, by combining data from Fiedler and Careau (2021) and Kermany et al. (2023), and evaluating the relationships between personality and (1) ‘willingness to run’ during the forced-exercise test, (2) metabolic scope and (3) the constituents of metabolic scope (i.e. RMR and *V̇*_O_2_,max_). Here, we analysed repeated behavioural and metabolic measurements using multivariate mixed models to partition correlations at the among- and within-individual levels. Our main objective was to test the idea that metabolic scope is ‘equally (or more)’ relevant to behaviour as RMR (as suggested by Biro et al., 2018).

## MATERIALS AND METHODS

### Ethics

All procedures were approved by the University of Ottawa Animal Care Committee under protocol #BLe-3227-A1, certified by the Canadian Council on Animal Care. All applicable international, national and/or institutional guidelines for the use of animals were followed.

### Study area and trapping

Data were collected at the Queens University Biological Station (44°34′08″N, 76°19′08″W) from May to October of 2018. For details, see Fiedler and Careau (2021). Briefly, mice, *Peromyscus leucopus* (Rafinesque 1818), were captured using Longworth-style traps set up on two separate grids (Cow Island, with 118 traps each placed 30 m apart; Blueberry Hill, with 64 traps also spaced 30 m apart). Traps were set at dusk and checked at dawn. Upon every capture, mice underwent a bag test immediately after their transfer from the trap to a meshed handling bag. The bag test is a simple, easy and widely popular test used to measure docility as the time spent immobile over the 60 s duration of the test (during which the handling bag is held at arm's length from the observer). After the bag test, a series of manipulations were done to mark or identify the mouse with ear tags, and determine its age (based on pelage), sex, reproductive status and body mass. Mice were then either released immediately or brought to an adjacent laboratory where they underwent either a 10 min open-field test (Caron-Lévesque and Careau, 2023; Kermany et al., 2023) or respirometry trials. Metabolic measurements were taken in the same laboratory as for the open-field tests, but never on the same capture (i.e. on different days). All mice were released at their capture location within a maximum of 12 h after their initial capture.

### Open-field tests

Details of the open-field tests can be found in Kermany et al. (2023). Each mouse was placed one at a time in an open circular plastic tank (diameter 140 cm) and was video-recorded under moderate lighting for 10 min by a camera above the arena. The arena was cleaned before and after each test to standardise conditions. Video-tracking software (EthovisionXT) was used to measure the distance moved, time spent grooming, number of jumps, duration in centre area, latency to enter centre area, meander score, maximum moving speed and total number of defaecations (urine and faecal boli combined) during the test. Distance moved, centre duration and latency to enter centre area were cubic-root transformed, whereas time spent grooming, number of jumps, number of defaecations and maximum speed were square-root transformed. Preliminary univariate mixed models were used to estimate repeatability of each open-field variable (Table S1), and only significantly repeatable variables were retained for further analysis.

### Metabolic measurements

Details of the respirometry protocols can be found in Fiedler and Careau (2021). Briefly, RMR was measured using an eight-channel open-flow respirometry system. Each mouse was placed into a cylindrical Plexiglas chamber (diameter 8 cm, length 15 cm) with a piece of flat metal mesh holding them above the Plexiglas. Each chamber was placed on a separate activity detector (ADX-C, Sable Systems International) which recorded movement within the chamber throughout the trial. Up to 8 mice could be measured at a time in our custom-built cabinet, which was temperature controlled to be within the thermal neutral zone of the mice (28°C) (Cannon and Nedergaard, 2011).

*V̇*_O_2_,max_ was measured using a pull-mode respirometry system that consisted of a Field Metabolic System (Sable Systems International) connected to a ∼2 l respirometry chamber which contained a treadmill with a 20 deg incline. Mice were placed in the chamber and the treadmill was started with speed increasing every 2 min. Mice were forced to exercise by using a metal grid (which emitted gentle electric pulses, 163 V, 0.5–1.5 mA, 200 ms, 1 pulse s^−1^) located at the end of the treadmill, such that mice that stopped running received mild electric shocks. The trials ended when the mouse rested on the metal grid for 10 s.

Following prior studies on performance (Swallow et al., 1998; Coleman et al., 1998; Chappell et al., 2007; Rezende et al., 2009; Le Galliard et al., 2013; Claghorn et al., 2017), we developed a semi-quantitative grading scale of the willingness or cooperativeness of the animal during the forced-exercise test. For example, Swallow et al. (1998) used a subjective assessment of run quality (5 categories from poor to excellent) in mice running on a treadmill. By contrast, Le Galliard et al. (2013) used a quantitative assessment of the motivation to run on a treadmill in common lizards (*Zootoca vivipara*) by counting the number of taps given on the animal during the forced-exercise tests. In our case, we used a combination of behavioural observations during and after the tests. Each trial was scored from 1 to 4 based on the willingness of the mouse to run during the trial. A score of 1 meant that the mouse did not run at all and showed normal behaviour after the trial. A score of 2 meant that the mouse ran but not consistently or vigorously. A score of 3 meant that the mouse ran vigorously but not consistently, while a score of 4 meant that the mouse ran both vigorously and consistently. For scores of both 3 and 4, the mouse showed signs of exhaustion after the end of the trial, and for this reason we consider that these trials yielded an appropriate measure of *V̇*_O_2_,max_. Hence, willingness to run was considered a behavioural variable when it included all forced-exercised trials and possible scores (1 to 4), but was considered a nuisance variable when it was restricted to trials that yielded an acceptable measure of *V̇*_O_2_,max_ (i.e. 3 and 4).

### Statistical analysis

We estimated the among-individual correlations (*r*_ind_) and the within-individual correlations (*r*_e_) using a total of three separate multivariate mixed models fitted with the package ASReml-R version 4 (Butler et al., 2018). In all models, individual identity was included as a random effect with an unstructured correlation structure which allowed for estimates of among-individual variance (*V*_ind_) and *r*_ind_. The residuals were also modelled with an unstructured correlation structure to get estimates of within-individual variance (*V*_e_) and *r*_e_.

We fitted a total of three separate multivariate mixed models to extract the correlations of interest. The first multivariate mixed model (model 1) was fitted with willingness to run, docility and all of the repeatable open-field traits as response variables. To analyse the relationship between metabolic scope and our behavioural traits, the second multivariate mixed model (model 2) was fitted with metabolic scope, docility and all of the repeatable open-field traits as response variables. Finally, to analyse the relationship between RMR, *V̇*_O_2_,max_ and the behavioural traits, a third multivariate mixed model (model 3) was fitted with the two metabolic and the same behavioural traits as model 2. Although this approach may seem redundant, each model provided a relevant piece of information that could not easily be obtained by fitting a single multivariate model that included all of the traits. First, we wanted to estimate the relationship between behavioural traits and willingness to run while considering all of the scores (1 to 4). In models 2 and 3, however, the scope and *V̇*_O_2_,max_ measures were restricted to forced-exercise trials that yielded scores of 3 or 4. Moreover, we wanted to statistically control for the slight difference in *V̇*_O_2_,max_ between trials that yielded a score of 3 versus 4 (as in Fiedler and Careau, 2021), but willingness to run cannot be fitted as both a dependent variable and a fixed effect (fitted to scope and *V̇*_O_2_,max_) in the same model. Note that we also re-ran models 2 and 3 without conditioning scope or *V̇*_O_2_,max_ on willingness to run, and it did not change any of our conclusions. Finally, scope cannot be included in the same model as RMR and *V̇*_O_2_,max_ because a multivariate model cannot include a variable that is a mathematical combination of other variables in the model. We therefore fitted a total of three separate multivariate mixed models and extracted the correlation of interest to reduce redundancy (e.g. the correlations among docility and the open-field variables were estimated in each model, but only those from model 1 are presented in Table S2).

All response variables were standardized (mean of zero and standard deviation of 1). Repeatability of willingness to run (model 1), metabolic scope (model 2) and RMR and *V̇*_O_2_,max_ (model 3) was calculated from this model, as *R*=*V*_ind_/(*V*_ind_+*V*_e_). All models included fixed effects of age (adult or juvenile), sex, reproductive status (yes or no), body mass, time of day, test sequence (how many times have they gone through the test before) and day of year fitted to all response variables. Some nuisance variables were also fitted as fixed effects when applicable (e.g. willingness to run was fitted as a fixed effect to scope and *V̇*_O_2_,max_ in models 2 and 3). Although some of our variables did not follow a gaussian distribution on the raw scale (i.e. willingness to run and docility), after correction for the many fixed effects included in the mixed models, the residuals did not substantially deviate from an approximated normal distribution, suggesting that our model estimates were robust (Schielzeth et al., 2020). We used likelihood ratio tests to estimate the significance of each *r*_ind_ and *r*_e_. The *r*_e_ between the open-field variables and forced-exercise measures (i.e. willingness to run, *V̇*_O_2_,max_ and metabolic scope) could not be estimated because the two traits were never measured on the same day.

R code (Script 1) and raw data (Dataset 1) are available in the supplementary information.

### Declaration of AI use

We have not used AI-assisted technologies in creating this article.

## RESULTS AND DISCUSSION

### Repeatability

The 153 individuals on which Fiedler and Careau (2021) measured RMR and *V̇*_O_2_,max_ were captured a total of 950 times in 2018. Docility was measured 902 times on 148 individuals, averaged 43 s (range: 0 to 60 s), and was significantly repeatable at *R*=0.272±0.041. Open-field tests were conducted 210 times on 97 individuals, and distance moved averaged 3489 cm (range: 1 to 21,578 cm). Many open-field variables were significantly repeatable, except number of jumps, latency to enter centre area and meander score (Table S1). As previously reported in Fiedler and Careau (2021), the repeatability of willingness to run and the metabolic traits was low but significant (willingness to run: *R*=0.196±0.062; metabolic scope: *R*=0.307±0.129; RMR: *R*=0.118±0.073; *V̇*_O_2_,max_: *R*=0.250±0.114). We note that these repeatability estimates are relatively low, which makes among-individual correlations more difficult to detect (see below). Still, our repeatability estimates for docility and the open-field traits fall relatively close to the average for behavioural traits (0.37; Bell et al., 2009). For the metabolic traits, our estimates are somewhat lower than the average, which may be due to the fact that this study was conducted on wild-caught individuals, which typically display lower repeatability of metabolic rate than laboratory animals (Auer et al., 2016).

### Behavioural traits and willingness to run (model 1)

At the among-individual level, willingness to run during the forced-exercise test was not significantly correlated with docility or the open-field traits (Table 1). The absence of among-individual correlations between the behavioural traits and willingness to run suggests that excluding the ‘low motivation’ trials did not bias our sampling based on personality of the individuals.

**Table 1.** **Among- and within-individual correlations between metabolic variables [willingness to run, metabolic scope, resting metabolic rate (RMR) and** ***V̇*****_O_2_,max_] and behavioural variables (docility and five open-field variables) in wild white-footed mice**

		Willingness to run	Metabolic scope	RMR	*V̇*_O_2_,max_
Among-individual correlations					
Docility		0.159±0.230	0.112±0.200	0.171±0.238	0.000±0.211
Distance moved		−0.133±0.297	−0.022±0.257	0.335±0.326	0.061±0.267
Time spent grooming		−0.202±0.341	−0.160±0.307	**0.832±0.352**	−0.154±0.306
Duration in centre		−0.253±0.348	−0.385±0.298	0.590±0.379	−0.371±0.314
Maximum speed		0.265±0.322	0.245±0.287	−0.044±0.355	0.439±0.293
Number of defecations		−0.039±0.316	−0.279±0.272	0.421±0.359	−0.172±0.294
Within-individual correlations					
Docility		**−0.141±0.069**	0.167±0.100	−0.029±0.067	0.137±0.093

Data are correlation estimates (*r*) ±s.e. Estimates presented in bold are statistically significant according to profile likelihoods.

**Fig. 1.**
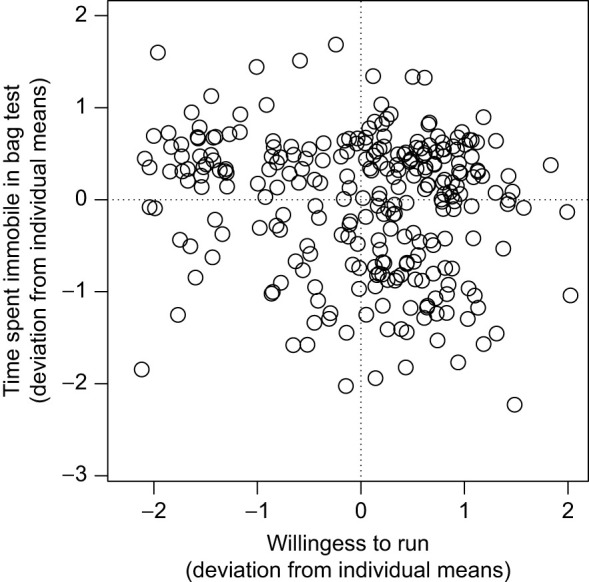
**Representation of the within-individual correlation (*r*_e_) between docility and willingness to run during a forced-exercise test in wild white-footed mice (*Peromyscus leucopus*).** Docility was measured as time spent immobile during a bag test. Deviations (in s.d. units) from individual means are displayed as the residuals extracted from multivariate model 1. Shown are *n*=269 paired observations made on *N=*130 individuals.

At the within-individual level, however, there was a significant negative correlation between willingness to run and docility (*r*_e_=−0.141±0.069; χ^2^_1_=4.0, *P*=0.045; Fig. 1). This negative *r*_e_ between docility and willingness to run implies that on days when mice moved more in the bag test (less docile), they also were more willing to run. Within-individual (residual) correlations can result from correlated measurement error or phenotypic plasticity (Dingemanse and Dochtermann, 2013). In our case, correlated measurement error is unlikely as docility and willingness to run were measured on two separate tests with differing scales. This means that the correlation is likely the result of phenotypic plasticity in response to one or more unknown environmental variables. Such environmental variables may cause within-individual variation in the freezing response of the mice, which in turn simultaneously affects their locomotor response to being held captive and prompted to run. Of the possible mechanisms worth exploring, perhaps the most likely to play a role would be stress-induced analgesia (SIA) (Marek and Szacki, 1988; Łapo et al. 2003). Indeed, if a mouse is more stressed than usual on a given day, it may freeze for longer than usual during the bag test, and subsequent SIA could reduce its response to the motivating stimuli during the forced-exercise test.

### Behavioural traits and metabolic scope (model 2)

At the among-individual level, metabolic scope was not significantly correlated with docility or the open-field traits (Table 1). At the within-individual level, however, metabolic scope tended to be positively correlated with docility (*r*_e_=0.167±0.100), but the correlation was marginally non-significant (χ^2^_1_=2.57, *P*=0.109). This trend, although non-significant, was positive, which is in the opposite direction to that predicted by Biro et al. (2018). Moreover, the relationship was almost entirely driven by *V̇*_O_2_,max_. A quick look at the raw variability in the metabolic traits makes it obvious that the variance in *V̇*_O_2_,max_ is much greater than in RMR (Fig. S1A). As a result, metabolic scope co-varies strongly with *V̇*_O_2_,max_ (Fig. S1B), but the correlation with RMR is much weaker (Fig. S1C). The almost perfect 1-to-1 relationship with *V̇*_O_2_,max_ implies that any variation we see in metabolic scope primarily reflects variation in *V̇*_O_2_,max_, suggesting that no additional information is added by using metabolic scope instead of both RMR and *V̇*_O_2_,max_ separately. Thus, our results highlight that care must be taken when interpreting relationships between metabolic scope and behavioural traits, because in many if not most cases the reasoning will be more parsimonious if it is based on *V̇*_O_2_,max_ rather than scope.

### Behavioural traits, RMR and *V̇*_O_2_,max_ (model 3)

Docility was not correlated with either *V̇*_O_2_,max_ or RMR at the among- or within-individual levels (Table 1). Of the open-field traits, only time spent grooming was significantly correlated with RMR at the among-individual level (Table 1; *r*_ind_=0.832±0.352; χ^2^_1_=4.07, *P*=0.043). Therefore, mice that had a higher RMR tended to spend a longer time grooming during the open-field test. One potential explanation for this relationship could be increased stress levels. RMR has been shown to positively relate to physiological stress (glucocorticoid hormones) across mammals and birds (Haase et al., 2016; but see Jimeno and Verhulst, 2023, for how to interpret these correlations). Grooming in rodents can be a stress-induced behaviour (Kalueff et al., 2016; Rojas-Carvajal and Brenes, 2020), so the correlation between time spent grooming and RMR could be the result of behavioural and physiological responses to stress, causing some individuals to groom more during the open-field test and having an elevated RMR. Further studies could test for this relationship by differentiating the type of grooming, as thoroughness or region-specific grooming may relate more or less to stress and therefore RMR (Rojas-Carvajal and Brenes, 2020).

**Fig. 2.**
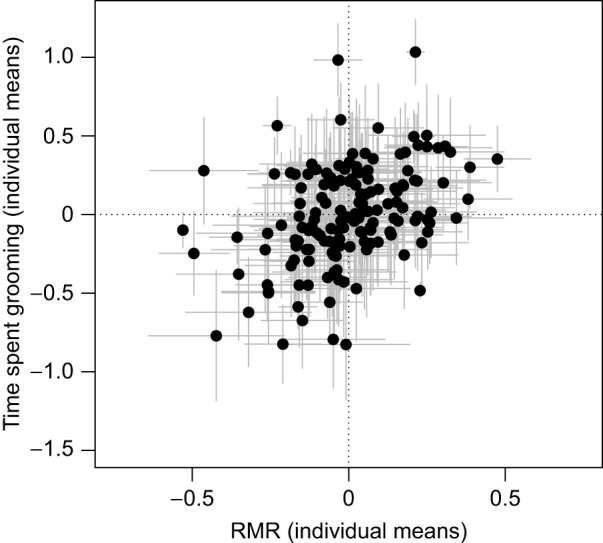
**Representation of the among-individual correlations (*r*_ind_) between time spent grooming in the open-field test and resting metabolic rate (RMR) in wild white-footed mice (*P. leucopus*).** Displayed are the individual means (±s.e.m., expressed as deviation from the population mean) extracted from multivariate model 3 with *z*-standardised traits (mean=0, variance=1). *N*=153 individuals.

Finally, we note that RMR tended to be positively correlated with distance moved and time spent in the centre area of the open-field test (Fig. 2), meaning that individuals that moved more in the open-field test tended to show higher RMR. These correlations, however, were not significant, which is not consistent with a past study on a closely related species (*Peromyscus maniculatus*) reporting a positive genetic correlation between distance moved and RMR (Careau et al., 2011). Moreover, a positive inter-specific correlation between distance moved and RMR was found when looking across seven species of the Neotominae family (Careau et al., 2009), which includes *P. maniculatus* and *leucopus*. While non-significant, the among-individual correlation we obtained for *P. leucopus* was estimated using data from only one year (i.e. the year when *V̇*_O_2_,max_ was measured at our field site). Future studies, with a larger sample size including more years and information on the genetic relatedness of individuals, will be needed to confirm whether distance moved and RMR are positively correlated at the genetic level as observed in the Neotominae family (Careau et al., 2009). A meta-analysis of relationships between RMR and multiple behaviours found no overall significant relationship when restricting the analysis to behaviours that are putatively weakly associated with net energy gain or loss (Mathot et al., 2019). Although exploration (proxied by distance moved and time spent in the centre in an open-field test) may be perceived as having little influence on energy gain or loss in most animals, this may not be the case for mice. Indeed, mice require high levels of activity and exploration to find unpredictable and dispersed high-value foods (Koteja and Weiner, 1993); thus, exploratory behaviour may be a key component of the energy budget of the mouse lifestyle.

## Supplementary Material

10.1242/jexbio.250374_sup1 Supplementary information

Dataset 1. Raw data
