# Supplementary material for: Personality and metabolic scope in wild mice
Source: J Exp Biol. 2025 Aug 11;228(15):jeb250374. doi: 10.1242/jeb.250374 (PMC12401533; doi:10.1242/jeb.250374)
Supplement: Supplementary information [file jexbio-228-250374-s1.pdf]

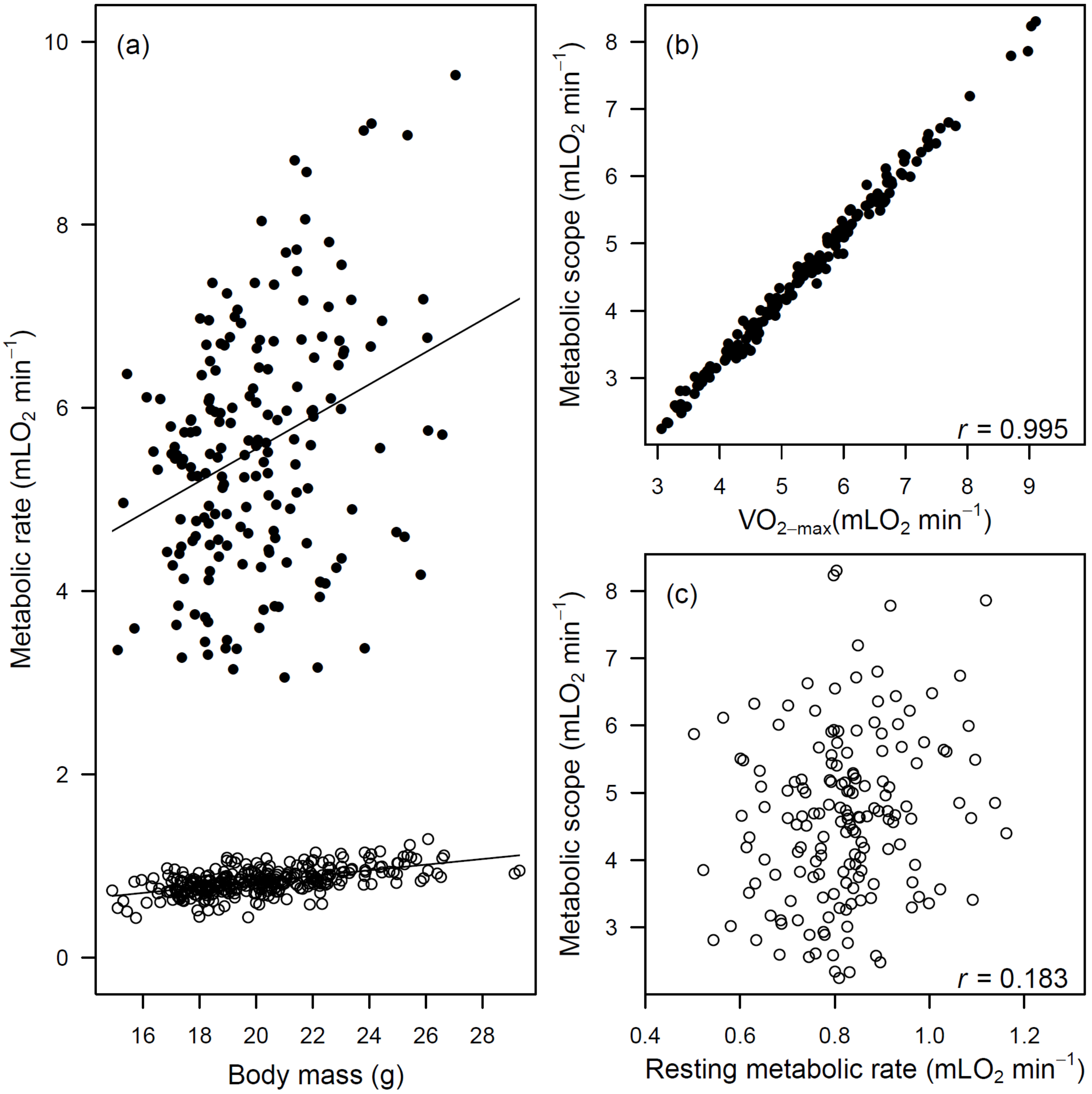

Fig. S1. (a) Metabolic rate (mLO$_2$ min^{-1}; open symbols = resting metabolic rate; closed symbols = $VO_{2\text{-}max}$) as function of body mass (g) in wild white-footed mice. Metabolic scope as function of (b) $VO_{2\text{-}max}$ and (c) resting metabolic rate in wild white-footed mice (*Peromyscus leucopus*).

Dataset 1. Raw data

Available for download at
https://journals.biologists.com/jeb/article-lookup/doi/10.1242/jeb.250374#supplementary-data

Journal of Experimental Biology • Supplementary information

Table S1. Among-individual variance (V_{ind}), residual variance (V_e), and repeatability (R) in eight variables extracted from the open-field tests conducted on wild white-footed mice. Only the five significantly repeatable variables (in bold) were retained in further analysis for testing relationships with metabolic rate.

Open-field variable	V_{ind} ± se	V_e ± se	R ± se	$\chi 2$	P
**Distance moved**	**0.235 ± 0.078**	**0.440 ± 0.060**	**0.348 ± 0.102**	**14.28**	**<0.001**
**Time spent grooming**	**0.168 ± 0.089**	**0.837 ± 0.103**	**0.167 ± 0.092**	**5.58**	**0.018**
Number of jumps	0.000 ± NA	0.914 ± 0.089	0.000 ± 0.000	0.00	1.000
**Time spent in center area**	**0.143 ± 0.072**	**0.642 ± 0.082**	**0.182 ± 0.093**	**6.38**	**0.012**
Latency to enter center area	0.152 ± 0.112	0.769 ± 0.122	0.165 ± 0.128	2.03	0.154
Meander score	0.061 ± 0.080	0.959 ± 0.119	0.059 ± 0.082	0.73	0.392
**Maximum speed**	**0.190 ± 0.090**	**0.674 ± 0.090**	**0.220 ± 0.105**	**5.89**	**0.015**
**Defecations**	**0.198 ± 0.088**	**0.725 ± 0.092**	**0.215 ± 0.097**	**8.25**	**0.004**

Table S2. Among-individual correlations (r_{ind}) and within-individual correlations (r_e) between docility (i.e., time spent immobile during a bag test) and the five repeatable variables extracted from the open-field tests.

		Among-individual correlations	Within-individual correlations
Trait 1	Trait 2	r_{ind} ± se	r_e ± se
Distance moved	Docility	-0.376 ± 0.166	0.021 ± 0.084
Time spent grooming	Docility	-0.552 ± 0.192	0.069 ± 0.079
Time spent grooming	Distance moved	0.339 ± 0.247	0.260 ± 0.084
Time spent in center area	Docility	-0.421 ± 0.205	-0.049 ± 0.081
Time spent in center area	Distance moved	0.829 ± 0.117	0.659 ± 0.051
Time spent in center area	Time spent grooming	0.436 ± 0.301	0.054 ± 0.088
Maximum speed	Docility	-0.334 ± 0.186	0.139 ± 0.084
Maximum speed	Distance moved	0.717 ± 0.142	0.482 ± 0.071
Maximum speed	Time spent grooming	0.763 ± 0.228	0.145 ± 0.087
Maximum speed	Time spent in center area	0.469 ± 0.266	0.141 ± 0.089
Defecations	Docility	-0.211 ± 0.188	0.117 ± 0.081
Defecations	Distance moved	0.989 ± 0.169	0.035 ± 0.090
Defecations	Time spent grooming	0.544 ± 0.246	0.196 ± 0.084
Defecations	Time spent in center area	0.820 ± 0.235	0.076 ± 0.088
Defecations	Maximum speed	0.831 ± 0.215	0.035 ± 0.089

Script 1

R code

```
rm(list=ls())
library(asreml)
library(nadiv)
load("DATA.RData")
################ descriptive statistics ##################
################ descriptive statistics ##################
################ descriptive statistics ##################
################ descriptive statistics ##################
nrow(DATA)
length(unique(DATA$ID))

#reproduce numbers in Fiedler and Careau Table 2
RMR_data<-DATA[!is.na(DATA$RMR),]
nrow(RMR_data)
length(unique(RMR_data$ID))
mean(RMR_data$RMR)
range(RMR_data$RMR)

MMR_data<-DATA[!is.na(DATA$MMR),]
nrow(MMR_data)
length(unique(MMR_data$ID))
mean(MMR_data$MMR)
range(MMR_data$MMR)

#descriptive stats for docility and exploration
BAG_data<-DATA[!is.na(DATA$BAG_TEST),]
nrow(BAG_data)
length(unique(BAG_data$ID))
mean(BAG_data$BAG_TEST)
range(BAG_data$BAG_TEST)

EXP<-DATA[!is.na(DATA$OF_dist),]
nrow(EXP)
length(unique(EXP$ID))
mean(EXP$OF_dist)
range(EXP$OF_dist)

#################### transform and re-scale variables ###########################
#################### transform and re-scale variables ###########################
```

Journal of Experimental Biology • Supplementary information

```
#################### transform and re-scale variables ###########################
#################### transform and re-scale variables ###########################
#################### transform and re-scale variables ###########################
#################### transform and re-scale variables ###########################
#################### transform and re-scale variables ###########################
DATA$JULIANz<-scale(DATA$JULIAN)
DATA$MeanTempz<-scale(DATA$MeanTemp)
DATA$RMRz<-scale(DATA$RMR)
DATA$ACTz<-scale(DATA$ACT_RMR)
DATA$MMRz<-scale(DATA$MMR)
DATA$BAGz<-scale(DATA$BAG_TEST)
DATA$MASSz<-scale(DATA$MASS_end)

#OF variables
DATA$GRMz<-scale(DATA$OF_GROOM_t^0.5)
DATA$JMPz<-scale(DATA$OF_JUMP^0.5)
DATA$CTRz<-scale(DATA$OF_CTR_DUR^(1/3))
DATA$TRNz<-scale(DATA$OF_TURN_ANGLE)
DATA$MEAz<-scale(DATA$OF_MEANDER)
DATA$DEFz<-scale((DATA$OF_caca+DATA$OF_pipi)^0.5)
DATA$OF_SPEED_max[which(DATA$OF_SPEED_max>300)]<-NA #delete one outlier
DATA$MAXz<-scale(DATA$OF_SPEED_max^0.5)
DATA$LATz<-scale(DATA$OF_CTR_LAT^(1/3))
DATA$EXPz<-scale(DATA$OF_dist^(1/3))

#calculate absolute scope
DATA$SCOPE <- NA
DATA$SCOPE <- (DATA$MMR - DATA
$RMR) plot(SCOPE~RMR,DATA)
plot(SCOPE~quality,DATA)
DATA$SCPz <- scale(DATA$SCOPE)

DATA$FACTORIAL <- NA
DATA$FACTORIAL <- (DATA$MMR/DATA$RMR)
plot(FACTORIAL~RMR,DATA)
plot(FACTORIAL~quality,DATA)
DATA$FACz <- scale(DATA$FACTORIAL)
##################### Figure S1 ################################
cor.test(DATA$SCOPE,DATA$RMR)
cor.test(DATA$SCOPE,DATA$MMR)
```

Journal of Experimental Biology • Supplementary information

```
DATA <-  DATA[order(DATA$RMR),]
DATA$sort <- seq(1:nrow(DATA))
var(DATA$RMR, na.rm=T)
var(DATA$MMR,na.rm=T)

par(las=1)
plot(MMR~sort,data=DATA, ylim=c(0,10),xlim=c(0,325),xlab="individual",pch=16)
points(MMR~sort,data=DATA,col=2)
points(RMR~sort,data=DATA)
abline(lm(MMR~sort,data=DATA))
abline(lm(RMR~sort,data=DATA))

par(mfrow=c(2,2),las=1,oma=c(1,1,1,1),mar=c(3,3,1,1))
layout(matrix(c(1,1,2,3),2,2))
layout.show(3)
plot(MMR~MASS_end,data=DATA, ylim=c(0,10),xlab="",ylab="",pch=16)
mtext(expression(paste("Metabolic rate (mLO"[2], " min"^-1,")")),side=2,las=3,line=2)
mtext("Body mass (g)",side=1,line=2.5)
mtext("(a)",side=3,line=-2,adj=0.05)
points(RMR~MASS_end,data=DATA)
clip(x1=min(DATA$MASS_end,na.rm=T),x2=max(DATA$MASS_end,na.rm=T),
y1=min(DATA$MMR,na.rm=T),y2=max(DATA$MMR,na.rm=T))
abline(lm(MMR~MASS_end,data=DATA))
clip(x1=min(DATA$MASS_end,na.rm=T),x2=max(DATA$MASS_end,na.rm=T),
y1=min(DATA$RMR,na.rm=T),y2=max(DATA$RMR,na.rm=T))
abline(lm(RMR~MASS_end,data=DATA))
#
plot(SCOPE~MMR,DATA,pch=16,ylab="",xlab="")
mtext("(b)",side=3,line=-2,adj=0.05)
mtext(expression(paste("VO"[2-max], "(mLO"[2]," min"^-1,")")),side=1,line=2.5)
mtext(expression(paste("Metabolic scope (mLO"[2], " min"^-1,")")),side=2,las=3,line=2)
mtext(expression(paste(italic("r")," = 0.995")),side=1,line=-1,adj=0.95)
#
plot(SCOPE~RMR,DATA,ylab="",xlab="")
mtext("(c)",side=3,line=-2,adj=0.05)
mtext(expression(paste("Resting metabolic rate (mLO"[2]," min"^-1,")")),side=1,line=2.5)
mtext(expression(paste(italic("r")," = 0.183")),side=1,line=-1,adj=0.95)


##################### repeatability of OF variables ############################
##################### repeatability of OF variables ############################
```

```
##################### repeatability of OF variables #############################
##################### repeatability of OF variables #############################
##################### repeatability of OF variables #############################
##################### repeatability of OF variables #############################
##################### repeatability of OF variables #############################
#univariate mixed models with ID random effect
GRM.M <-
asreml(GRMz~MASS_FIELDz+AGEc+SEXc+PARAc+REPc+JULIANz+MeanTempz+OF_TIME_MIN+S
EQ_OF,random=~ID,data=DATA, na.action=na.method(x="include"))
JMP.M <-
asreml(JMPz~MASS_FIELDz+AGEc+SEXc+PARAc+REPc+JULIANz+MeanTempz+OF_TIME_MIN+SE
Q_OF,random=~ID,data=DATA, na.action=na.method(x="include"))
CTR.M <-
asreml(CTRz~MASS_FIELDz+AGEc+SEXc+PARAc+REPc+JULIANz+MeanTempz+OF_TIME_MIN+SE
Q_OF,random=~ID,data=DATA, na.action=na.method(x="include"))
LAT.M <-
asreml(LATz~MASS_FIELDz+AGEc+SEXc+PARAc+REPc+JULIANz+MeanTempz+OF_TIME_MIN+SE
Q_OF,random=~ID,data=DATA, na.action=na.method(x="include"))
MEA.M <-
asreml(MEAz~MASS_FIELDz+AGEc+SEXc+PARAc+REPc+JULIANz+MeanTempz+OF_TIME_MIN+S
EQ_OF,random=~ID,data=DATA, na.action=na.method(x="include"))
MAX.M <-
asreml(MAXz~MASS_FIELDz+AGEc+SEXc+PARAc+REPc+JULIANz+MeanTempz+OF_TIME_MIN+S
EQ_OF,random=~ID,data=DATA, na.action=na.method(x="include"))
DEF.M <-
asreml(DEFz~MASS_FIELDz+AGEc+SEXc+PARAc+REPc+JULIANz+MeanTempz+OF_TIME_MIN+SE
Q_OF,random=~ID,data=DATA, na.action=na.method(x="include"))
EXP.M <-
asreml(EXPz~MASS_FIELDz+AGEc+SEXc+PARAc+REPc+JULIANz+MeanTempz+OF_TIME_MIN+SE
Q_OF,random=~ID,data=DATA, na.action=na.method(x="include"))
#calculate repeatability
(R.GRM<-vpredict(GRM.M, R~V1/(V1+V2)))
(R.JMP<-vpredict(JMP.M, R~V1/(V1+V2)))
(R.CTR<-vpredict(CTR.M, R~V1/(V1+V2)))
(R.LAT<-vpredict(LAT.M, R~V1/(V1+V2)))
(R.MEA<-vpredict(MEA.M, R~V1/(V1+V2)))
(R.MAX<-vpredict(MAX.M, R~V1/(V1+V2)))
(R.DEF<-vpredict(DEF.M, R~V1/(V1+V2)))
(R.EXP<-vpredict(EXP.M, R~V1/(V1+V2)))

#reduced model without ID random effect
```

Journal of Experimental Biology • Supplementary information

```
GRM.r <-
asreml(GRMz~MASS_FIELDz+AGEc+SEXc+PARAc+REPc+JULIANz+MeanTempz+OF_TIME_MIN+S
EQ_OF,data=DATA, na.action=na.method(x="include"))
JMP.r <-
asreml(JMPz~MASS_FIELDz+AGEc+SEXc+PARAc+REPc+JULIANz+MeanTempz+OF_TIME_MIN+SE
Q_OF,data=DATA, na.action=na.method(x="include"))
CTR.r <-
asreml(CTRz~MASS_FIELDz+AGEc+SEXc+PARAc+REPc+JULIANz+MeanTempz+OF_TIME_MIN+SE
Q_OF,data=DATA, na.action=na.method(x="include"))
LAT.r <-
asreml(LATz~MASS_FIELDz+AGEc+SEXc+PARAc+REPc+JULIANz+MeanTempz+OF_TIME_MIN+SE
Q_OF,data=DATA, na.action=na.method(x="include"))
MEA.r <-
asreml(MEAz~MASS_FIELDz+AGEc+SEXc+PARAc+REPc+JULIANz+MeanTempz+OF_TIME_MIN+S
EQ_OF,data=DATA, na.action=na.method(x="include"))
MAX.r <-
asreml(MAXz~MASS_FIELDz+AGEc+SEXc+PARAc+REPc+JULIANz+MeanTempz+OF_TIME_MIN+S
EQ_OF,data=DATA, na.action=na.method(x="include"))
DEF.r <-
asreml(DEFz~MASS_FIELDz+AGEc+SEXc+PARAc+REPc+JULIANz+MeanTempz+OF_TIME_MIN+SE
Q_OF,data=DATA, na.action=na.method(x="include"))
EXP.r <-
asreml(EXPz~MASS_FIELDz+AGEc+SEXc+PARAc+REPc+JULIANz+MeanTempz+OF_TIME_MIN+SE
Q_OF,data=DATA, na.action=na.method(x="include"))
(CHI.GRM<-2*(GRM.M$loglik-GRM.r$loglik))
(CHI.JMP<-2*(JMP.M$loglik-JMP.r$loglik))
(CHI.CTR<-2*(CTR.M$loglik-CTR.r$loglik))
(CHI.LAT<-2*(LAT.M$loglik-LAT.r$loglik))
(CHI.MEA<-2*(MEA.M$loglik-MEA.r$loglik))
(CHI.MAX<-2*(MAX.M$loglik-MAX.r$loglik))
(CHI.DEF<-2*(DEF.M$loglik-DEF.r$loglik))
(CHI.EXP<-2*(EXP.M$loglik-EXP.r$loglik))
(P.GRM<-1-pchisq(CHI.GRM,df=1))
(P.JMP<-1-pchisq(CHI.JMP,df=1))
(P.CTR<-1-pchisq(CHI.CTR,df=1))
(P.LAT<-1-pchisq(CHI.LAT,df=1))
(P.MEA<-1-pchisq(CHI.MEA,df=1))
(P.MAX<-1-pchisq(CHI.MAX,df=1))
(P.DEF<-1-pchisq(CHI.DEF,df=1))
(P.EXP<-1-pchisq(CHI.EXP,df=1))
#make Table S1
```

```
Table.S1<-rbind(c(summary(EXP.M)$varcomp[1,1:2], summary( EXP.M)$varcomp[2,1:2],
R.EXP,CHI.EXP,P.EXP),
                    c(summary(GRM.M)$varcomp[1,1:2], summary(GRM.M)$varcomp[2,1:2],
R.GRM,CHI.GRM,P.GRM),
                    c(summary(JMP.M)$varcomp[1,1:2], summary(JMP.M)$varcomp[2,1:2],
R.JMP,CHI.JMP,P.JMP),
                    c(summary(CTR.M)$varcomp[1,1:2], summary(CTR.M)$varcomp[2,1:2],
R.CTR,CHI.CTR,P.CTR),
                    c(summary(LAT.M)$varcomp[1,1:2], summary(LAT.M)$varcomp[2,1:2],
R.LAT,CHI.LAT,P.LAT),
                    c(summary(MEA.M)$varcomp[1,1:2], summary(MEA.M)$varcomp[2,1:2],
R.MEA,CHI.MEA,P.MEA),
                    c(summary(MAX.M)$varcomp[1,1:2], summary(MAX.M)$varcomp[2,1:2],
R.MAX,CHI.MAX,P.MAX),
                    c(summary(DEF.M)$varcomp[1,1:2], summary(DEF.M)$varcomp[2,1:2],
    R.DEF,CHI.DEF,P.DEF))
Table.S1

#################### Willingness to Run Stats #####################
#################### Willingness to Run Stats #####################
#################### Willingness to Run Stats #####################
#################### Willingness to Run Stats #####################
#################### Willingness to Run Stats #####################
#################### Willingness to Run Stats #####################
#################### Willingness to Run Stats #####################
#################### Willingness to Run Stats #####################
#################### Willingness to Run Stats #####################
#################### Willingness to Run Stats #####################
temp<- asreml(cbind(BAGz,EXPz,GRMz,CTRz,MAXz,DEFz,quality)~trait,
                  random=~corgh(trait):ID+at(trait,1):HANDLER_FIELD,
                  residual=~units:corgh(trait),
                  data=DATA, na.action=na.method(x="include"),
                  start.values=T)
TRI.start<-temp$vparameters TRI.start
$Value[47:51]<-0
TRI.start$Constraint[47:51]<-"F"

Tri.M <- asreml(cbind(BAGz,EXPz,GRMz,CTRz,MAXz,DEFz,quality)~trait+
                    at(trait):MASS_FIELDz+
                    at(trait):AGEc+
                    at(trait):SEXc+
```

```
              at(trait):PARAc+
              at(trait):REPc+
              at(trait):JULIANz+
              at(trait):MeanTempz+
              at(trait,7):TIME_MMR_MIN+
              at(trait,1):TIME_BAG_MIN+
              at(trait,2:6):OF_TIME_MIN+
              at(trait,7):SEQM+
              at(trait,1):SEQ_FIELD+
              at(trait,2:6):SEQ_OF,
              random=~corgh(trait):ID+at(trait,1):HANDLER_FIELD,
             residual=~units:corgh(trait),
             data=DATA, na.action=na.method(x="include"), maxiter=100,
             R.param=TRI.start)

Tri.M<-update.asreml(Tri.M) Tri.M
$conv
summary(Tri.M)

#repeatability of willingess to run
vpredict(Tri.M, R~V28/(V28+V58)) #
Estimate        SE
#R 0.1958807 0.06163726

#likelihood ratio test
TRI.redu<-TRI.start
TRI.redu$Value[46]<-0
TRI.redu$Constraint[46]<-"F"

redu.tri<-asreml(cbind(BAGz,EXPz,GRMz,CTRz,MAXz,DEFz,quality)~trait+
              at(trait):MASS_FIELDz+
              at(trait):AGEc+
              at(trait):SEXc+
              at(trait):PARAc+
              at(trait):REPc+
              at(trait):JULIANz+
              at(trait):MeanTempz+
              at(trait,7):TIME_MMR_MIN+
              at(trait,1):TIME_BAG_MIN+
              at(trait,2:6):OF_TIME_MIN+
              at(trait,7):SEQM+
```

Journal of Experimental Biology • Supplementary information

```
          at(trait,1):SEQ_FIELD+
          at(trait,2:6):SEQ_OF,
          random=~corgh(trait):ID+at(trait,1):HANDLER_FIELD,
         residual=~units:corgh(trait),
         data=DATA, na.action=na.method(x="include"), maxiter=100,
         R.param=TRI.redu)

summary(redu.tri)
(CHI.tri<-2*(Tri.M$loglik-redu.tri$loglik))
(P.tri<-1-pchisq(CHI.tri,df=1))

#make Table 2?
PROe.BAG.WILL<-
proLik4(Tri.M,component="units:trait!trait!quality:!trait!BAGz.cor",G=F,negative=T)
plot(PROe.BAG.WILL)
PROe.BAG.WILL
#$UCL
#[1] -0.04377231
#
#$LCL
#[1] -0.235086

#extract residuals to make Figure S1
length(resid(Tri.M))
DATA$res.bag <-resid(Tri.M)[seq(1, 6650, by = 7)]
DATA$res.exp <-resid(Tri.M)[seq(2, 6650, by = 7)]
DATA$res.grm <-resid(Tri.M)[seq(3, 6650, by = 7)]
DATA$res.ctr <-resid(Tri.M)[seq(4, 6650, by = 7)]
DATA$res.max <-resid(Tri.M)[seq(5, 6650, by = 7)]
DATA$res.def <-resid(Tri.M)[seq(6, 6650, by = 7)]
DATA$res.qua <-resid(Tri.M)[seq(7, 6650, by = 7)]
#check to see if the correct residual values were assigned to the correct rows
DATA[,c("quality","res.qua","BAGz","res.bag","EXPz","res.exp")]

par(mfrow=c(3,2))
hist(DATA$res.qua, main="",xlab="quality (model residuals)")
qqnorm(DATA$res.qua, main="")
qqline(DATA$res.qua)
#
hist(DATA$res.bag, main="",xlab="docility (model residuals)")
qqnorm(DATA$res.bag, main="")
```

Journal of Experimental Biology • Supplementary information

```
qqline(DATA$res.bag)
#
hist(DATA$res.exp, main="",xlab="exploration (model residuals)")
qqnorm(DATA$res.exp, main="")
qqline(DATA$res.exp)

#####################  Figure 1 ########################################
cor.test(DATA$res.qua,DATA$res.bag)
x11(5,5)
par(mar=c(5,5,1,1), las=1)
plot(res.bag~res.qua,DATA,xlab="",ylab="",cex=1.5,pch=1)
abline(v=0, lty=3)
abline(h=0, lty=3)
mtext("Time spent immobile in bag test",   cex=1.25,side=2, line=3,las=3)
mtext("(deviations from individual means)",cex=1.25,side=2, line=2,las=3)
mtext("Willingess to run",                 cex=1.25,side=1, line=2.0)
mtext("(deviations from individual means)",cex=1.25,side=1, line=3)

temp1<-DATA[!is.na(DATA$res.bag),]
temp2<-temp1[!is.na(temp1$res.qua),]
nrow(temp2)
temp2$ID<-factor(temp2$ID)
unique(temp2$ID)
################### Metabolic scope Stats ####################
################### Metabolic scope Stats ####################
################### Metabolic scope Stats ####################
################### Metabolic scope Stats ####################
################### Metabolic scope Stats ####################
################### Metabolic scope Stats ####################
################### Metabolic scope Stats #################### TEMP
<- asreml(cbind(BAGz,EXPz,GRMz,CTRz,MAXz,DEFz,SCPz)~trait,
                random=~corgh(trait):ID+at(trait,1):HANDLER_FIELD,
                residual=~units:corgh(trait),
                data=DATA, na.action=na.method(x="include"), maxiter=100,
                start.values=T)
start.values.SCP<-TEMP$vparameters
start.values.SCP$Value[47:51]<-0
start.values.SCP$Constraint[47:51]<-"F"

SCP.M <- asreml(cbind(BAGz,EXPz,GRMz,CTRz,MAXz,DEFz,SCPz)~trait+
                 at(trait,7):MASSz+
```

Journal of Experimental Biology • Supplementary information

```
                at(trait,1:6):MASS_FIELDz+
                at(trait):AGEc+
                at(trait):SEXc+
                at(trait):PARAc+
                at(trait):REPc+
                #at(trait,1):TIME_RMR_MIN+
                at(trait,7):TIME_MMR_MIN+
                at(trait,1):TIME_BAG_MIN+
                at(trait,2:6):OF_TIME_MIN+
                #at(trait,1):SEQR+
                at(trait,7):SEQM+
                at(trait,1):SEQ_FIELD+
                at(trait,2:6):SEQ_OF+
                at(trait):JULIANz+
                at(trait):MeanTempz+
                at(trait,7):ACT_RMR+
                #at(trait,7):quality+
                at(trait,7):FMS,
               random=~corgh(trait):ID+at(trait,1):HANDLER_FIELD,
               residual=~units:corgh(trait),
               data=DATA, na.action=na.method(x="include"), maxiter=100,
               R.param=start.values.SCP)

nrow(DATA)
summary(SCP.M)

#repeatability of metabolic scope
vpredict(SCP.M, R~V28/(V28+V57)) #
Estimate        SE
#R 0.3073368 0.1285925

#likelihood ratio test
SCP.redu<-start.values.SCP SCP.redu
$Value[46]<-0
SCP.redu$Constraint[46]<-"F"

redu.SCP<-asreml(cbind(BAGz,EXPz,GRMz,CTRz,MAXz,DEFz,SCPz)~trait+
                at(trait,7):MASSz+
                at(trait,1:6):MASS_FIELDz+
                at(trait):AGEc+
                at(trait):SEXc+
```

Journal of Experimental Biology • Supplementary information

```
          at(trait):PARAc+
          at(trait):REPc+
          #at(trait,1):TIME_RMR_MIN+
          at(trait,7):TIME_MMR_MIN+
          at(trait,1):TIME_BAG_MIN+
          at(trait,2:6):OF_TIME_MIN+
          #at(trait,1):SEQR+
          at(trait,7):SEQM+
          at(trait,1):SEQ_FIELD+
          at(trait,2:6):SEQ_OF+
          at(trait):JULIANz+
          at(trait):MeanTempz+
          at(trait,7):ACT_RMR+
          #at(trait,7):quality+
          at(trait,7):FMS,
         random=~corgh(trait):ID+at(trait,1):HANDLER_FIELD,
         residual=~units:corgh(trait),
         data=DATA, na.action=na.method(x="include"), maxiter=100,
         R.param=SCP.redu)

summary(redu.SCP)
(CHI.SCP<-2*(SCP.M$loglik-redu.SCP$loglik))
(P.SCP<-1-pchisq(CHI.SCP,df=1))


#PRO.1<-proLik4(SCP.M,component="trait:ID!trait!SCPz:!trait!BAGz.cor",G=T,negative=T)
#PRO.2<-proLik4(SCP.M,component="trait:ID!trait!SCPz:!trait!EXPz.cor",G=T,negative=T)
#PRO.3<-proLik4(SCP.M,component="trait:ID!trait!SCPz:!trait!GRMz.cor",G=T,negative=T)
#PRO.4<-proLik4(SCP.M,component="trait:ID!trait!SCPz:!trait!CTRz.cor",G=T,negative=T)
#PRO.5<-proLik4(SCP.M,component="trait:ID!trait!SCPz:!trait!MAXz.cor",G=T,negative=T)
#PRO.6<-proLik4(SCP.M,component="trait:ID!trait!SCPz:!trait!DEFz.cor",G=T,negative=T)
#plot(PRO.4)
#PRO.4
#PRO<-proLik4(SCP.M,component="units:trait!trait!SCPz:!trait!BAGz.cor",G=F,negative=T)
#plot(PRO)
#PRO
#$UCL
#[1] 0.2882212
#
#$LCL
#[1] 0.004270319
```

```
#extract residuals to make Figure X
slength(resid(SCP.M))
DATA$res.bag2 <-resid(SCP.M)[seq(1, 6650, by = 7)]
DATA$res.exp2 <-resid(SCP.M)[seq(2, 6650, by = 7)]
DATA$res.grm  <-resid(SCP.M)[seq(3, 6650, by = 7)]
DATA$res.ctr  <-resid(SCP.M)[seq(4, 6650, by = 7)]
DATA$res.max  <-resid(SCP.M)[seq(5, 6650, by = 7)]
DATA$res.def  <-resid(SCP.M)[seq(6, 6650, by = 7)]
DATA$res.scp  <-resid(SCP.M)[seq(7, 6650, by = 7)]
#check to see if the correct residual values were assigned to the correct rows
DATA[,c("SCPz","res.scp","BAGz","res.bag2","EXPz","res.exp2")]

par(mfrow=c(3,2))
hist(DATA$res.scp, main="",xlab="metabolic scope (model residuals)")
qqnorm(DATA$res.scp, main="")
qqline(DATA$res.scp)
#
hist(DATA$res.bag2, main="",xlab="docility (model residuals)")
qqnorm(DATA$res.bag2, main="")
qqline(DATA$res.bag2)
#
hist(DATA$res.exp2, main="",xlab="exploration (model residuals)")
qqnorm(DATA$res.exp2, main="")
qqline(DATA$res.exp2)

#####################  Figure 1 ########################################
x11(5,5)
par(mar=c(5,5,1,1), las=1)
plot(res.bag2~res.scp,DATA,xlab="",ylab="",cex=1.5,pch=1,ylim=c(-2.5,1.5)) abline(v=0,
lty=3)
abline(h=0, lty=3)
mtext("Time spent immobile in bag test",   cex=1.25,side=2, line=3,las=3)
mtext("(deviations from individual means)",cex=1.25,side=2, line=2,las=3)
mtext("Metabolic scope",                   cex=1.25,side=1, line=2.0)
mtext("(deviations from individual means)",cex=1.25,side=1, line=3)

length(DATA$res.bag2[!is.na(DATA$res.bag2)])
length(DATA$res.scp[!is.na(DATA$res.scp)])
length(unique(DATA$ID[!is.na(DATA$res.scp)]))
```

Journal of Experimental Biology • Supplementary information

```
########### RMR, MMR, quality and docility and exploration ###############
########### RMR, MMR, quality and docility and exploration ###############
########### RMR, MMR, quality and docility and exploration ###############
########### RMR, MMR, quality and docility and exploration ############### 5
########### RMR, MMR, quality and docility and exploration ###############
########### RMR, MMR, quality and docility and exploration ###############
########### RMR, MMR, quality and docility and exploration ###############
########### RMR, MMR, quality and docility and exploration ############### temp
<- asreml(cbind(BAGz,EXPz,GRMz,CTRz,MAXz,DEFz,RMRz,MMRz)~trait,
                    random=~corgh(trait):ID,
                    residual=~units:corgh(trait),
                    data=DATA,
                    na.action=na.method(x="include"),
                    maxiter=100,start.values=T)
start.values<-temp$vparameters
start.values$Value[54:58]<-0
start.values$Value[60:64]<-0
start.values$Constraint[54:58]<-"F"
start.values$Constraint[60:64]<-"F"

    Quad.M <- asreml(cbind(BAGz,EXPz,GRMz,CTRz,MAXz,DEFz,RMRz,MMRz)~trait+
                       #at(trait,8):quality+
                       at(trait,7:8):MASSz+
                       at(trait,1:6):MASS_FIELDz+
                       at(trait):AGEc+
                       at(trait):SEXc+
                       at(trait):PARAc+
                       at(trait):REPc+
                       at(trait,7):TIME_RMR_MIN+
                       at(trait,8):TIME_MMR_MIN+
                       at(trait,1):TIME_BAG_MIN+
                       at(trait,2:6):OF_TIME_MIN+
                       at(trait,7):SEQR+
                       at(trait,8):SEQM+
                       at(trait,1):SEQ_FIELD+
                       at(trait,2:6):SEQ_OF+
                       at(trait):JULIANz+
                       at(trait):MeanTempz+
```

Journal of Experimental Biology • Supplementary information

```
            at(trait,7):ACT_RMR+
            at(trait,8):FMS,
           random=~corgh(trait):ID,
           residual=~units:corgh(trait),
           data=DATA,
           na.action=na.method(x="include"),
           maxiter=100,G.param=start.values,R.param=start.values)

Quad.M<-update.asreml(Quad.M)
wald.asreml(Quad.M,denDF = "default",ssType = "conditional")$Wald
summary(Quad.M)

#repeatability of RMR
vpredict(Quad.M, R~V35/(V35+V72)) #
Estimate        SE
#R 0.1183216 0.07322902


#repeatability of VO2max
vpredict(Quad.M, R~V36/(V36+V73)) #
Estimate        SE
#R 0.2500082 0.1141221

#likelihood ratio tests
start.values.bag.rmr<-start.values
start.values.exp.rmr<-start.values
start.values.grm.rmr<-start.values
start.values.ctr.rmr<-start.values
start.values.max.rmr<-start.values
start.values.def.rmr<-start.values
start.values.bag.rmr$Value[16]<-0
start.values.exp.rmr$Value[17]<-0
start.values.grm.rmr$Value[18]<-0
start.values.ctr.rmr$Value[19]<-0
start.values.max.rmr$Value[20]<-0
start.values.def.rmr$Value[21]<-0
start.values.bag.rmr$Constraint[16]<-"F"
start.values.exp.rmr$Constraint[17]<-"F"
start.values.grm.rmr$Constraint[18]<-"F"
start.values.ctr.rmr$Constraint[19]<-"F"
start.values.max.rmr$Constraint[20]<-"F"
```

Journal of Experimental Biology • Supplementary information

```
start.values.def.rmr$Constraint[21]<-"F"

redu.bag.quad<-asreml(cbind(BAGz,EXPz,GRMz,CTRz,MAXz,DEFz,RMRz,MMRz)~trait+
                        #at(trait,8):quality+
                        at(trait,7:8):MASSz+
                        at(trait,1:6):MASS_FIELDz+
                        at(trait):AGEc+
                        at(trait):SEXc+
                        at(trait):PARAc+
                        at(trait):REPc+
                        at(trait,7):TIME_RMR_MIN+
                        at(trait,8):TIME_MMR_MIN+
                        at(trait,1):TIME_BAG_MIN+
                        at(trait,2:6):OF_TIME_MIN+
                        at(trait,7):SEQR+
                        at(trait,8):SEQM+
                        at(trait,1):SEQ_FIELD+
                        at(trait,2:6):SEQ_OF+
                        at(trait):JULIANz+
                        at(trait):MeanTempz+
                        at(trait,7):ACT_RMR+
                        at(trait,8):FMS,
                      random=~corgh(trait):ID,
                      residual=~units:corgh(trait),
                      data=DATA,
                      na.action=na.method(x="include"),
                      maxiter=15,
                      G.param=start.values.bag.rmr,R.param=start.values.bag.rmr)

redu.exp.quad<-asreml(cbind(BAGz,EXPz,GRMz,CTRz,MAXz,DEFz,RMRz,MMRz)~trait+
                        #at(trait,8):quality+
                        at(trait,7:8):MASSz+
                        at(trait,1:6):MASS_FIELDz+
                        at(trait):AGEc+
                        at(trait):SEXc+
                        at(trait):PARAc+
                        at(trait):REPc+
                        at(trait,7):TIME_RMR_MIN+
                        at(trait,8):TIME_MMR_MIN+
                        at(trait,1):TIME_BAG_MIN+
                        at(trait,2:6):OF_TIME_MIN+
```

Journal of Experimental Biology • Supplementary information

```
 at(trait,7):SEQR+
 at(trait,8):SEQM+
 at(trait,1):SEQ_FIELD+
 at(trait,2:6):SEQ_OF+
 at(trait):JULIANz+
 at(trait):MeanTempz+
 at(trait,7):ACT_RMR+
 at(trait,8):FMS,
random=~corgh(trait):ID,
residual=~units:corgh(trait),
data=DATA,
na.action=na.method(x="include"),
maxiter=15,
G.param=start.values.exp.rmr,R.param=start.values.exp.rmr)

redu.grm.quad<-asreml(cbind(BAGz,EXPz,GRMz,CTRz,MAXz,DEFz,RMRz,MMRz)~trait+
 #at(trait,8):quality+
 at(trait,7:8):MASSz+
 at(trait,1:6):MASS_FIELDz+
 at(trait):AGEc+
 at(trait):SEXc+
 at(trait):PARAc+
 at(trait):REPc+
 at(trait,7):TIME_RMR_MIN+
 at(trait,8):TIME_MMR_MIN+
 at(trait,1):TIME_BAG_MIN+
 at(trait,2:6):OF_TIME_MIN+
 at(trait,7):SEQR+
 at(trait,8):SEQM+
 at(trait,1):SEQ_FIELD+
 at(trait,2:6):SEQ_OF+
 at(trait):JULIANz+
 at(trait):MeanTempz+
 at(trait,7):ACT_RMR+
 at(trait,8):FMS,
random=~corgh(trait):ID,
residual=~units:corgh(trait),
data=DATA,
na.action=na.method(x="include"),
maxiter=15,
G.param=start.values.grm.rmr,R.param=start.values.grm.rmr)
```

Journal of Experimental Biology • Supplementary information

```
redu.ctr.quad<-asreml(cbind(BAGz,EXPz,GRMz,CTRz,MAXz,DEFz,RMRz,MMRz)~trait+
                #at(trait,8):quality+
                at(trait,7:8):MASSz+
                at(trait,1:6):MASS_FIELDz+
                at(trait):AGEc+
                at(trait):SEXc+
                at(trait):PARAc+
                at(trait):REPc+
                at(trait,7):TIME_RMR_MIN+
                at(trait,8):TIME_MMR_MIN+
                at(trait,1):TIME_BAG_MIN+
                at(trait,2:6):OF_TIME_MIN+
                at(trait,7):SEQR+
                at(trait,8):SEQM+
                at(trait,1):SEQ_FIELD+
                at(trait,2:6):SEQ_OF+
                at(trait):JULIANz+
                at(trait):MeanTempz+
                at(trait,7):ACT_RMR+
                at(trait,8):FMS,
               random=~corgh(trait):ID,
               residual=~units:corgh(trait),
               data=DATA,
               na.action=na.method(x="include"),
               maxiter=15,
               G.param=start.values.ctr.rmr,R.param=start.values.grm.rmr)

redu.max.quad<-asreml(cbind(BAGz,EXPz,GRMz,CTRz,MAXz,DEFz,RMRz,MMRz)~trait+
                #at(trait,8):quality+
                at(trait,7:8):MASSz+
                at(trait,1:6):MASS_FIELDz+
                at(trait):AGEc+
                at(trait):SEXc+
                at(trait):PARAc+
                at(trait):REPc+
                at(trait,7):TIME_RMR_MIN+
                at(trait,8):TIME_MMR_MIN+
                at(trait,1):TIME_BAG_MIN+
                at(trait,2:6):OF_TIME_MIN+
                at(trait,7):SEQR+
```

Journal of Experimental Biology • Supplementary information

```
                  at(trait,8):SEQM+
                  at(trait,1):SEQ_FIELD+
                  at(trait,2:6):SEQ_OF+
                  at(trait):JULIANz+
                  at(trait):MeanTempz+
                  at(trait,7):ACT_RMR+
                  at(trait,8):FMS,
                 random=~corgh(trait):ID,
                 residual=~units:corgh(trait),
                 data=DATA,
                 na.action=na.method(x="include"),
                 maxiter=15,
                 G.param=start.values.max.rmr,R.param=start.values.max.rmr)

redu.def.quad<-asreml(cbind(BAGz,EXPz,GRMz,CTRz,MAXz,DEFz,RMRz,MMRz)~trait+
                 # at(trait,8):quality+
                  at(trait,7:8):MASSz+
                  at(trait,1:6):MASS_FIELDz+
                  at(trait):AGEc+
                  at(trait):SEXc+
                  at(trait):PARAc+
                  at(trait):REPc+
                  at(trait,7):TIME_RMR_MIN+
                  at(trait,8):TIME_MMR_MIN+
                  at(trait,1):TIME_BAG_MIN+
                  at(trait,2:6):OF_TIME_MIN+
                  at(trait,7):SEQR+
                  at(trait,8):SEQM+
                  at(trait,1):SEQ_FIELD+
                  at(trait,2:6):SEQ_OF+
                  at(trait):JULIANz+
                  at(trait):MeanTempz+
                  at(trait,7):ACT_RMR+
                  at(trait,8):FMS,
                 random=~corgh(trait):ID,
                 residual=~units:corgh(trait),
                 data=DATA,
                 na.action=na.method(x="include"),
                 maxiter=15,
                 G.param=start.values.def.rmr,R.param=start.values.def.rmr)
```

Journal of Experimental Biology • Supplementary information

```
(chi.bag.rmr<-2*(Quad.M$loglik-redu.bag.quad$loglik))
(chi.exp.rmr<-2*(Quad.M$loglik-redu.exp.quad$loglik))
(chi.grm.rmr<-2*(Quad.M$loglik-redu.grm.quad$loglik))
(chi.ctr.rmr<-2*(Quad.M$loglik-redu.ctr.quad$loglik))
(chi.max.rmr<-2*(Quad.M$loglik-redu.max.quad$loglik))
(chi.def.rmr<-2*(Quad.M$loglik-redu.def.quad$loglik))
(P.bag<-1-pchisq(chi.bag.rmr,df=1))
(P.exp<-1-pchisq(chi.exp.rmr,df=1))
(P.grm<-1-pchisq(chi.grm.rmr,df=1))
(P.ctr<-1-pchisq(chi.ctr.rmr,df=1))
(P.max<-1-pchisq(chi.max.rmr,df=1))
(P.def<-1-pchisq(chi.def.rmr,df=1))


#PRO.BAG.RMR<-
proLik4(Quad.M,component="trait:ID!trait!RMRz:!trait!BAGz.cor",G=T,negative=T)
#PRO.EXP.RMR<-
proLik4(Quad.M,component="trait:ID!trait!RMRz:!trait!EXPz.cor",G=T,negative=T)
#PRO.GRM.RMR<-
proLik4(Quad.M,component="trait:ID!trait!RMRz:!trait!GRMz.cor",G=T,negative=T)
#PRO.CTR.RMR<-
proLik4(Quad.M,component="trait:ID!trait!RMRz:!trait!CTRz.cor",G=T,negative=T)
#PRO.MAX.RMR<-
proLik4(Quad.M,component="trait:ID!trait!RMRz:!trait!MAXz.cor",G=T,negative=T)
#PRO.DEF.RMR<-
proLik4(Quad.M,component="trait:ID!trait!RMRz:!trait!DEFz.cor",G=T,negative=T)
#PRO.BAG.MMR<-
proLik4(Quad.M,component="trait:ID!trait!MMRz:!trait!BAGz.cor",G=T,negative=T)
#PRO.EXP.MMR<-
proLik4(Quad.M,component="trait:ID!trait!MMRz:!trait!EXPz.cor",G=T,negative=T)
#PRO.GRM.MMR<-
proLik4(Quad.M,component="trait:ID!trait!MMRz:!trait!GRMz.cor",G=T,negative=T)
#PRO.CTR.MMR<-
proLik4(Quad.M,component="trait:ID!trait!MMRz:!trait!CTRz.cor",G=T,negative=T)
#PRO.MAX.MMR<-
proLik4(Quad.M,component="trait:ID!trait!MMRz:!trait!MAXz.cor",G=T,negative=T)
#PRO.DEF.MMR<-
proLik4(Quad.M,component="trait:ID!trait!MMRz:!trait!DEFz.cor",G=T,negative=T)
#PRO.BAG.RMR
#PRO.EXP.RMR
#PRO.GRM.RMR
```

Journal of Experimental Biology • Supplementary information

```
#PRO.CTR.RMR
#PRO.MAX.RMR
#PRO.DEF.RMR
#PRO.BAG.MMR
#PRO.EXP.MMR
#PRO.GRM.MMR
#PRO.CTR.MMR
#PRO.MAX.MMR
#PRO.DEF.MMR
#


#extract residuals to make Figure S2
length(resid(Quad.M))
DATA$res.bag3 <-resid(Quad.M)[seq(1, 7600, by = 8)]
DATA$res.exp3 <-resid(Quad.M)[seq(2, 7600, by = 8)]
DATA$res.grm3 <-resid(Quad.M)[seq(3, 7600, by = 8)]
DATA$res.ctr3 <-resid(Quad.M)[seq(4, 7600, by = 8)]
DATA$res.max3 <-resid(Quad.M)[seq(5, 7600, by = 8)]
DATA$res.def3 <-resid(Quad.M)[seq(6, 7600, by = 8)]
DATA$res.rmr3 <-resid(Quad.M)[seq(7, 7600, by = 8)]
DATA$res.mmr3 <-resid(Quad.M)[seq(8, 7600, by = 8)]
#check to see if the correct residual values were assigned to the correct rows
DATA[,c("SCPz","res.rmr3","BAGz","res.bag3","EXPz","res.exp3")]


x11()
par(mfrow=c(4,2))
hist(DATA$res.rmr3, main="",xlab="resting metabolic rate (model residuals)")
qqnorm(DATA$res.rmr3, main="")
qqline(DATA$res.rmr3)
#
hist(DATA$res.mmr3, main="",xlab=expression(paste("VO"[2-max], "(model residual)", sep = " ")))
qqnorm(DATA$res.mmr3, main="")
qqline(DATA$res.mmr3)
#
hist(DATA$res.bag3, main="",xlab="docility (model residuals)")
qqnorm(DATA$res.bag3, main="")
qqline(DATA$res.bag3)
#
```

Journal of Experimental Biology • Supplementary information

```
hist(DATA$res.exp3, main="",xlab="exploration (model residuals)")
qqnorm(DATA$res.exp3, main="")
qqline(DATA$res.exp3)

cbind(sqrt(Quad.M$vcoeff$random*Quad.M$sigma2),(Quad.M$vcoeff$random*Quad.M$sigm a2))


###################################### BLUPS FIG 2 and GROOM fig

DvsS<-data.frame(Trait = rownames(Quad.M$coefficients$random),
                 BLUP = Quad.M$coefficients$random,
                 SE = sqrt(Quad.M$vcoeff$random*Quad.M$sigma2))
DvsS$ID<-substr(DvsS$Trait, 15,18)
DvsS$TRAIT<-substr(DvsS$Trait, 7,9)
DvsS$Trait<-NULL
colnames(DvsS)[1]<-"BLUP"
BLUPS<-reshape(DvsS, v.names = c("BLUP","SE"), idvar = "ID",timevar = "TRAIT", direction =
"wide")
nrow(BLUPS)
write.csv(BLUPS, file="BLUPS.csv", row.names=F)

x11(5,4.5)
par(mar=c(4,5,1,1))
plot(BLUP.GRM~BLUP.RMR,BLUPS,
        xlab="",
        ylab="",
        xlim=c(-0.75,0.75), ylim=c(-1.5,1.25), las=1, bty="o", col="red",cex.axis=1,cex.lab=1)
arrows(x0=BLUPS$BLUP.RMR,       y0=BLUPS$BLUP.GRM-
BLUPS$SE.GRM,x1=BLUPS$BLUP.RMR,
y1=BLUPS$BLUP.GRM+BLUPS$SE.GRM,code=3,angle=90,length=0,col="grey")
arrows(x0=BLUPS$BLUP.RMR-BLUPS$SE.RMR,y0=BLUPS$BLUP.GRM,
x1=BLUPS$BLUP.RMR+BLUPS$SE.RMR,y1=BLUPS$BLUP.GRM,code=3,angle=90,length=0,col="gr ey")
points(BLUP.GRM~BLUP.RMR,BLUPS, cex=1,pch=19,col="black")
abline(v=0,lty=3)
abline(h=0,lty=3)
mtext("Resting metabolic rate (individual means)",side=1,line=2)
mtext("Time spent grooming (individual means)",side=2,line=3.5,las=3)


plot(BLUP.EXP~BLUP.RMR,BLUPS,
        xlab="",
```

Journal of Experimental Biology • Supplementary information

```
        ylab="",
        xlim=c(-0.75,0.75), ylim=c(-1.5,1.25), las=1, bty="o", col="red",cex.axis=1,cex.lab=1)
arrows(x0=BLUPS$BLUP.RMR,          y0=BLUPS$BLUP.EXP-
BLUPS$SE.EXP,x1=BLUPS$BLUP.RMR,
y1=BLUPS$BLUP.EXP+BLUPS$SE.EXP,code=3,angle=90,length=0,col="grey")
arrows(x0=BLUPS$BLUP.RMR-BLUPS$SE.RMR,y0=BLUPS$BLUP.EXP,
x1=BLUPS$BLUP.RMR+BLUPS$SE.RMR,y1=BLUPS$BLUP.EXP,code=3,angle=90,length=0,col="gre y")
points(BLUP.EXP~BLUP.RMR,BLUPS, cex=1,pch=19,col="black")
abline(v=0,lty=3)
abline(h=0,lty=3)
mtext(expression(paste(italic("r")[ind]," = 0.41?0.31")),side=1, line=-1, adj=0.95,cex=1)
mtext("Resting metabolic rate (individual means)",side=1,line=2)
mtext("Distance moved (individual means)",side=2,line=3.5,las=3)


plot(BLUP.CTR~BLUP.RMR,BLUPS,
        xlab="",
        ylab="",
        xlim=c(-0.75,0.75), ylim=c(-1.5,1.25), las=1, bty="o", col="red",cex.axis=1,cex.lab=1)
arrows(x0=BLUPS$BLUP.RMR,          y0=BLUPS$BLUP.CTR-
BLUPS$SE.GRM,x1=BLUPS$BLUP.RMR,
y1=BLUPS$BLUP.CTR+BLUPS$SE.CTR,code=3,angle=90,length=0,col="grey")
arrows(x0=BLUPS$BLUP.RMR-BLUPS$SE.RMR,y0=BLUPS$BLUP.CTR,
x1=BLUPS$BLUP.RMR+BLUPS$SE.RMR,y1=BLUPS$BLUP.CTR,code=3,angle=90,length=0,col="gre y")
points(BLUP.CTR~BLUP.RMR,BLUPS, cex=1,pch=19,col="black")
abline(v=0,lty=3)
abline(h=0,lty=3)
mtext(expression(paste(italic("r")[ind]," = 0.41?0.31")),side=1, line=-1, adj=0.95,cex=1)
mtext("Resting metabolic rate (individual means)",side=1,line=2)
mtext("Time spent in center area (individual means)",side=2,line=3.5,las=3)


cor.test(BLUPS$BLUP.GRM,BLUPS$BLUP.RMR)
cor.test(BLUPS$BLUP.CTR,BLUPS$BLUP.RMR)
cor.test(BLUPS$BLUP.EXP,BLUPS$BLUP.RMR)

#BAG RMR correlation
plot(BLUP.BAG~BLUP.RMR,BLUPS,
        xlab="",
        ylab="",
        xlim=c(-0.75,0.75), ylim=c(-1.5,1.25), las=1, bty="o", col="red",cex.axis=1.2,cex.lab=1.5)
```

Journal of Experimental Biology • Supplementary information

```
arrows(x0=BLUPS$BLUP.RMR,y0=BLUPS$BLUP.BAG-
BLUPS$SE.BAG,x1=BLUPS$BLUP.RMR,y1=BLUPS$BLUP.BAG+BLUPS$SE.BAG,code=3,angle=90,le
ngth=0)
arrows(x0=BLUPS$BLUP.RMR-
BLUPS$SE.RMR,y0=BLUPS$BLUP.BAG,x1=BLUPS$BLUP.RMR+BLUPS$SE.RMR,y1=BLUPS$BLUP.B
AG,code=3,angle=90,length=0)
points(BLUP.BAG~BLUP.RMR,BLUPS, cex=1,pch=19,col="red")
abline(v=0,lty=3)
abline(h=0,lty=3)
#mtext(expression(paste(italic("R")[ind]," = 0.460Â±0.30")),side=3, line=0.25, adj=0.5,cex=1)

#GRM RMR correlation
x11(5,5)
par(mar=c(4,5,1,1))
plot(BLUP.GRM~BLUP.RMR,BLUPS,
        xlab="",
        ylab="",
        xlim=c(-0.75,0.75), ylim=c(-1.5,1.25), las=1, bty="o", col="red",cex.axis=1,cex.lab=1)
arrows(x0=BLUPS$BLUP.RMR,           y0=BLUPS$BLUP.GRM-
BLUPS$SE.GRM,x1=BLUPS$BLUP.RMR,
y1=BLUPS$BLUP.GRM+BLUPS$SE.GRM,code=3,angle=90,length=0,col="grey")
arrows(x0=BLUPS$BLUP.RMR-BLUPS$SE.RMR,y0=BLUPS$BLUP.GRM,
x1=BLUPS$BLUP.RMR+BLUPS$SE.RMR,y1=BLUPS$BLUP.GRM,code=3,angle=90,length=0,col="gr ey")
points(BLUP.GRM~BLUP.RMR,BLUPS, cex=1,pch=19,col="black")
abline(v=0,lty=3)
abline(h=0,lty=3)
mtext(expression(paste(italic("r")[ind]," = 0.55Â±0.38")),side=1, line=-1, adj=0.95,cex=1)
mtext("Resting metabolic rate (individual means)",side=1,line=2)
mtext("Time spent grooming (individual means)",side=2,line=3.5,las=3)
```
